# Auditory clicks elicit equivalent temporal frequency perception to tactile pulses: A cross-modal psychophysical study

**DOI:** 10.3389/fnins.2022.1006185

**Published:** 2022-09-09

**Authors:** Deepak Sharma, Kevin K. W. Ng, Ingvars Birznieks, Richard M. Vickery

**Affiliations:** ^1^School of Biomedical Sciences, The University of New South Wales (UNSW Sydney), Sydney, NSW, Australia; ^2^Neuroscience Research Australia, Sydney, NSW, Australia; ^3^Center for Social and Affective Neuroscience, Department of Biomedical and Clinical Sciences, Linköping University, Linköping, Sweden; ^4^Bionics and Bio-Robotics, Tyree Foundation Institute of Health Engineering, The University of New South Wales (UNSW Sydney), Sydney, NSW, Australia

**Keywords:** vibrotactile, audio-tactile equivalence, burst-gap code, temporal code, auditory click, frequency perception, cross-modal comparison, pitch perception

## Abstract

Both hearing and touch are sensitive to the frequency of mechanical oscillations—sound waves and tactile vibrations, respectively. The mounting evidence of parallels in temporal frequency processing between the two sensory systems led us to directly address the question of perceptual frequency equivalence between touch and hearing using stimuli of simple and more complex temporal features. In a cross-modal psychophysical paradigm, subjects compared the perceived frequency of pulsatile mechanical vibrations to that elicited by pulsatile acoustic (click) trains, and vice versa. Non-invasive pulsatile stimulation designed to excite a fixed population of afferents was used to induce desired temporal spike trains at frequencies spanning flutter up to vibratory hum (>50 Hz). The cross-modal perceived frequency for regular test pulse trains of either modality was a close match to the presented stimulus physical frequency up to 100 Hz. We then tested whether the recently discovered “burst gap” temporal code for frequency, that is shared by the two senses, renders an equivalent cross-modal frequency perception. When subjects compared trains comprising pairs of pulses (bursts) in one modality against regular trains in the other, the cross-sensory equivalent perceptual frequency best corresponded to the silent interval between the successive bursts in both auditory and tactile test stimuli. These findings suggest that identical acoustic and vibrotactile pulse trains, regardless of pattern, elicit equivalent frequencies, and imply analogous temporal frequency computation strategies in both modalities. This perceptual correspondence raises the possibility of employing a cross-modal comparison as a robust standard to overcome the prevailing methodological limitations in psychophysical investigations and strongly encourages cross-modal approaches for transmitting sensory information such as translating pitch into a similar pattern of vibration on the skin.

## Introduction

The frequency of environmental mechanical oscillations is sampled by two human sensory systems, auditory and tactile. Notwithstanding that these signals are transduced through quite different physical media and separate sensory epithelia, there are a surprising number of physiological commonalities between them (von Békésy, 1959; Saal et al., 2016). Both require the mechanical displacement of frequency-tuned receptors to transduce physical events into neural signals, and both modalities generate temporally-precise spiking responses in their primary afferents capable of conveying rapid time-varying signals (Saal et al., 2016). Furthermore, in both sensory modalities, low-frequency stimuli (<50 Hz) elicit a sensation of flutter where individual stimulus pulses are discriminable (Pollack, 1952), while high-frequency stimuli (>50 Hz) evoke a sensation of vibratory hum or pitch where stimulus pulses fuse into a singular or a continuous percept (Talbot et al., 1968; Krumbholz et al., 2000). The range of frequencies detectable by the skin (2–1,000 Hz) is noted to overlap partially with that sensed by the ear (20–20,000 Hz) (Bolanowski et al., 1988).

Temporal frequency analysis is a fundamental part of sensory processing in both modalities: in audition, temporal frequency analysis is required for perception of speech and music (Javel and Mott, 1988); in touch, it is essential for perception of surface texture (Mackevicius et al., 2012) and sensing of the environment through hand-held tools (Brisben et al., 1999). The relative first spike latencies across primary afferents are used to locate sound sources in audition (Grothe et al., 2010), and in touch to determine features of objects, such as curvature and force direction upon first contact (Johansson and Birznieks, 2004). Though sensitive to different stimulus energies, these two sensory modalities may implement similar coding strategies to extract behaviourally relevant stimulus information (Pack and Bensmaia, 2015).

Aside from the correspondences between auditory and tactile processing of environmental vibrations, various behavioural experiments have demonstrated reciprocal perceptual interactions between the two sensory modalities, suggesting an intimate link in the perception of frequency signals (Occelli et al., 2011). For example, concurrent acoustic stimuli have been shown to influence the detection of tactile vibrations (Wilson et al., 2010), the perception of vibrotactile frequency (Ro et al., 2009; Yau et al., 2009a), and even the perception of surface texture (Guest et al., 2002). Reciprocally, simultaneous tactile cues influenced auditory pitch perception (Convento et al., 2019), loudness perception (Yau et al., 2010), and thereby, speech comprehension (Gick and Derrick, 2009). The observed behavioural interactions are further substantiated by the evidence of neuronal responses in the sensory cortices to stimuli not of their principal modality. The human auditory cortex has been shown to respond to tactile stimulation (Foxe et al., 2002; Kayser et al., 2005; Schürmann et al., 2006), and reciprocally, human participants performing an auditory frequency discrimination task demonstrated auditory frequency representation responses distributed over somatosensory cortical areas (Pérez-Bellido et al., 2018; Rahman et al., 2020). Additionally, lesions to the somatosensory cortex in rats were found to systematically impact the processing of auditory information in the auditory cortex (Escabí et al., 2007), corroborating the evidence of anatomical projections connecting the two brain regions (Ro et al., 2013).

The similarities in temporal frequency processing, the significant cross-sensory influence on frequency perception, and the evidence of neural responses in the alternate sensory cortex during stimulus presentation, invite the possibility of perceptual frequency equivalence between touch and hearing. The question has not previously been directly addressed, but in light of the parallels outlined above, we sought to investigate whether a perceptual equivalence of frequency could be achieved between auditory and tactile pulse stimuli. For instance, what would be the auditory equivalence of a 50 Hz vibrotactile pulse train and vice versa? And can cross-sensory comparison stimuli be used to assess the perceived frequency of stimuli delivered to the other sensory modality? A demonstrated equivalency of perceptual frequency may permit cross-modal methods for transmitting sensory information, for example, translating pitch into a similar pattern of vibration on the skin (Marks, 1983). The ability to use an alternate sense as a standard in psychophysical experiments opens new experimental possibilities.

In this study, we aimed to determine whether perceived frequency of pulsatile mechanical vibrations can be matched with that elicited by acoustic pulse (click) trains, and vice versa by performing cross-modal psychophysical frequency discrimination experiments. In these experiments, we first tested stimuli spanning the flutter and vibratory hum range in their simplest form (regular acoustic and vibrotactile pulse trains). Second, we compared stimuli organised as bursts of pulses in one modality with regular stimuli in the other, with the hypothesis that the frequency of the matching regular pulse train will equal that predicted from the inter-burst gap, as previously shown in unimodal studies (Birznieks and Vickery, 2017; Ng et al., 2020, 2021; Sharma et al., 2022). The pulsatile stimulation technique employed in this study allows precise control of the timing of spikes in a fixed population of primary afferents responding to the stimulus modality. Each mechanical and acoustic pulse is a repeatable and uniform event, ensuring that the same population of afferents is activated regardless of how frequently these pulses are repeated (Vickery et al., 2020).

## Materials and methods

The study was a controlled laboratory experiment involving behavioural measurements of the ability of human subjects to discriminate frequencies between vibrotactile and acoustic pulse trains. The participants performed a two-alternative forced-choice (2AFC) task where they discriminated which of two sequentially presented audio-tactile stimuli in a pair was perceived as higher in frequency. These judgements were recorded by a button press.

### Subjects

Twelve healthy subjects (aged 19–42, six females) without any known history or presenting clinical signs of auditory and somatosensory disorders, screened *via* questionnaire, volunteered in the study. The experimental protocols were approved by the Human Research Ethics Committee of UNSW Sydney (HC210271), and written informed consent was obtained from all the subjects before conducting the experiments.

### Mechanical pulse train generation and delivery

The required pulse trains, always of 1 s duration, were generated using custom scripts written in MATLAB (MathWorks, Natick, MA, United States) and Spike2 (Cambridge Electronic Design, Cambridge, United Kingdom) software. The generated stimulus waveforms (pulse trains) were converted to analogue voltage signals using a CED Power1401 mk II (Cambridge Electronic Design, Cambridge, United Kingdom), and then amplified by a PA 100E Power Amplifier (Data physics, San Jose, CA, United States) to drive the SignalForce GW-V4 shaker (Data Physics, San Jose, CA, United States). A probe with a 5 mm diameter metal ball attached to its tip delivered the mechanical pulses to the fingertip skin along the axis perpendicular to the skin surface. An OptoNCDT 2200–10 laser displacement sensor (Micro-Epsilon, Ortenburg, Germany) with a resolution of 0.3 μm at 10 kHz was used to detect the displacement of the stimulation probe.

Stimuli were delivered to the finger pad of the right index finger (dominant hand) which was secured to a finger rest. The participant’s forearm was immobilised with the aid of a GermaProtec vacuum pillow (AB Germa, Kristianstad, Sweden) which was moulded around the arm and deflated to maintain its shape and hold the arm in position. The probe was positioned to contact the skin with a pre-indentation force of around 0.1 N, as determined by the calibrated displacement of the probe from the laser recordings. The stimulus site was approximately halfway between the distal interphalangeal joint and the fingertip. The unstimulated fingers of the tested hand were not in contact with any surface.

Each vibrotactile pulse was a reproducible and uniform event with a protraction time <2 ms. With the pulse duration being comparable to the refractory period of an action potential, each mechanical stimulation event elicited only a single time-controlled spike in responding afferents, which was previously validated by microneurographic recordings (Birznieks and Vickery, 2017). The amplitude of each mechanical pulse vibration was set to 32 μm so as to activate both fast adapting type I (FAI) and fast adapting type II (FAII) afferents (Birznieks et al., 2019; Ng et al., 2022). Any sound produced by the shaker was masked using white noise delivered *via* headphones to the subjects.

### Acoustic pulse train generation and delivery

Like the vibrotactile stimuli, 1 s acoustic pulse trains of desired temporal characteristics were generated using MATLAB (MathWorks, Natick, MA, United States) and Spike2 (Cambridge Electronic Design, Cambridge, United Kingdom) software and converted to analogue voltage signals using a Power 1401 (CED, Cambridge, United Kingdom). The output signal was fed to a Vonyx STM500BT 2-channel mixer (Tronios, Netherlands), where it was mixed with white noise before delivery binaurally *via* wired Bose QuietComfort 35 noise-cancelling headphones (Bose, United States) to the subjects. The auditory stimuli were presented against a background of continuous white noise, with great care taken to ensure that the auditory clicks remained salient and easily perceivable.

Each acoustic pulse was a 1 ms, fixed amplitude, Gaussian-modulated 5 kHz sinewave designed to excite a fixed population of cochlear afferents, thereby precluding place-cues code for pitch perception (Sharma et al., 2022). This atypical auditory stimulation technique allows participants to hear pulsatile qualities akin to vibrotactile stimulations.

### Psychophysical experiment: Audio-tactile cross-modal frequency discrimination

**FIGURE 1:**
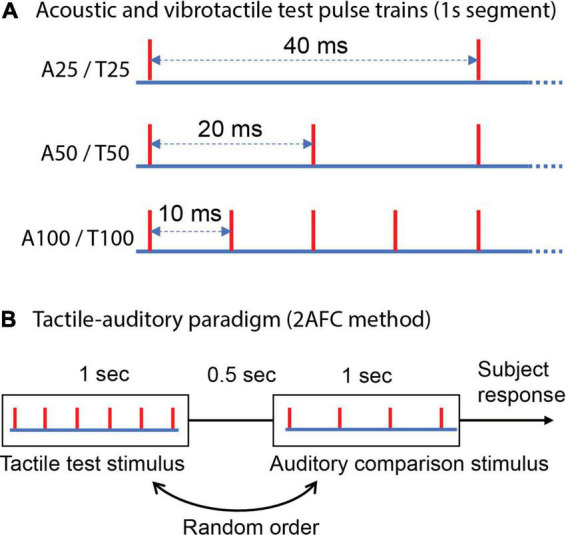
Schematic representation of test stimuli and the experimental protocol. **(A)** Auditory and vibrotactile 1 s regular test pulse trains (evenly spaced individual pulses); “A” denotes auditory and “T” tactile; a subsequent number indicates the frequency. Each red vertical line indicates the time of a mechanical pulse or an acoustic pulse. **(B)** Two alternative forced-choice (2AFC) method used to estimate cross-modal equivalent or matched frequency for test stimuli, tactile-auditory paradigm is presented as an example [i.e., a tactile test pulse train compared with six different regular acoustic pulse trains spanning a range of frequencies to obtain an auditory point of subjective equality (PSE)].

A 2AFC method was used to determine the perceptually equivalent frequency in one sensory modality for a test stimulus delivered to the other modality (Figure 1B). Each test stimulus (acoustic or tactile) was compared against six different regular pulse trains (trains of evenly spaced individual pulses) delivered to the other sensory system in a cross-modal manner (i.e., for a tactile test stimulus, acoustic pulse trains were used, and vice versa). This gave us two psychophysical experiment paradigms: auditory-tactile, where a test is auditory, with tactile stimuli as comparisons, and similarly tactile-auditory paradigm.

We matched the perceived intensity of a tactile pulse and an acoustic pulse (on the white noise background) for each subject so that the attention shift across modalities would not be dominated by one type. This was achieved by first finding a white noise volume for each subject that completely masked sounds from the vibrotactile shaker. Then subjects were presented trains of auditory clicks at 25 Hz of varying voltage and asked to compare intensity with that of tactile taps presented at 25 Hz and a fixed amplitude of 32 μm.

A pair of 1 s stimuli was delivered on each trial—a test and one of the six cross-modal comparison trains selected randomly, presented in random order and separated by 0.5 s. The subject indicated which of the two stimuli had the higher perceived frequency by button press. Subjects’ responses were acquired by the Power1401 and recorded in Spike2 for further analysis. Continuous white noise was delivered *via* the same headphones used for presenting auditory stimuli throughout the trials (including the duration of the two intervals and the 500 ms duration between intervals) to mask any auditory cues from the operation of the vibrotactile stimulation equipment. Subjects were instructed to ignore changes in the perceptual quality, and loudness or intensity elicited by the pulse trains if such changes were to occur and focus specifically on the frequency or repetition rate during frequency judgement. The participants practiced the cross-modal frequency discrimination task with a tactile test 25 Hz regular train compared five times against each of four regular acoustic pulse trains to ensure they understood the directions and could perform the task. No feedback was provided to the subjects during both the practice trials and the experimental trials.

We designed two experiments, one comparing regular trains of pulses, and one comparing paired pulses with regular comparison frequencies. This yielded 12 test blocks: tactile or auditory as test; with regular or paired as test; and test frequencies (25, 50, and 100 Hz). All blocks were tested on a given subject on 1 day and presented in random order. Subjects were given a short break after every three blocks.

To obtain psychometric curves, each test stimulus was compared 20 times against each of the six cross-modal comparison stimuli, giving rise to 120 trials per test condition. The 120 trials were randomised within each test condition and between subjects. For each comparison frequency, the proportion of times the participant responded that it was higher in frequency than the test was calculated (P_*H*_). The logit transformation ln(P_*H*_/(1-P_*H*_)) was then applied to the data to obtain a linear psychometric function. The frequency value at the zero crossing of the logit axis by the regression line fitted to the logit transformed data gave the point of subjective equality (PSE), which is the comparison frequency equally likely to be judged higher or lower than the test stimulus. This PSE value corresponds to the equivalent perceived or a matched frequency in the other modality for a given test stimulus. Discrimination sensitivity was measured by the Weber fraction, which was taken as one-half of the difference between the 25% point and the 75% on the psychometric function, divided by the frequency of the test stimulus (LaMotte and Mountcastle, 1975).

#### Experiment 1: Regular stimuli

This experiment investigated the cross-modal perceptual frequency equivalence between simple auditory and tactile stimuli of regular pulses.

A cross-modal frequency discrimination paradigm was used to assess the matching or equivalent frequency across audition and touch. The test stimuli were regular vibrotactile and acoustic 1 s pulse trains of 25, 50, and 100 Hz (Figure 1A). A PSE derived using auditory regular comparisons (which we term the auditory PSE) gives a matched/equivalent perceived frequency of an acoustic pulse train for a tactile test stimulus (which we called a tactile-auditory paradigm, Figure 1B). Similarly, a tactile PSE measures the tactile equivalence of an acoustic pulse train (auditory-tactile paradigm). The cross-modal regular comparison stimuli ranged from 12 to 44, 24–88, and 48–176 Hz for 25, 50, and 100 Hz test stimuli, respectively, regardless of the sensory modality used for the comparison. We compared how cross-modally achieved PSE differs from the expected unimodal perceived frequency which would equal the physical frequency for the regular train.

#### Experiment 2: Doublet burst patterns

This experiment investigated the perceptual frequency equivalence between complex temporal stimuli delivered in one modality and regular stimuli in the other.

**FIGURE 2:**
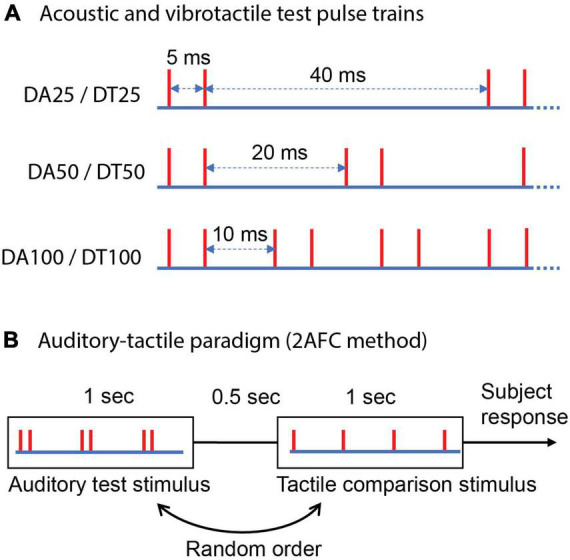
Schematic representation of test stimuli and the experimental procedure. **(A)** Acoustic and vibrotactile 1 s test pulse trains consisting of periodic bursts of two pulses spaced 5 ms apart. Each red vertical line indicates the time of a mechanical pulse or an acoustic pulse. “DA” denotes doublet auditory and “DT” doublet tactile; a subsequent number indicates frequency that is reciprocal of the inter-burst interval in a train. Test trains differ in the duration between two consecutive bursts. **(B)** Two alternative forced-choice (2AFC) method used to estimate cross-modal equivalent frequency, auditory-tactile paradigm is presented as an example.

In this experiment, we had subjects match the perceptual frequency between bursting stimuli in one sensory modality (tactile or auditory) and regular stimuli in the other modality. The bursting stimuli were 1 s pulse trains with periodic bursts of two pulses (doublet pattern). The PSE was determined using cross-modal regular trains, employing the same 2AFC method as in the first set of experiments (differing in test stimulus patterns, Figure 2B). The three doublet test stimuli are schematically illustrated in Figure 2A, and were identical for auditory and tactile. Each vertical line represents the timing of an acoustic pulse (auditory test) or a mechanical pulse (tactile test). Based on experimental results that help define the time envelope of a burst in the tactile system (Birznieks and Vickery, 2017; Ng et al., 2018, 2021), the two pulses in a burst were spaced 5 ms apart in all the test trains.

The inter-burst intervals in test trains were set at 40, 20, and 10 ms to correspond to expected perceived frequencies of 25, 50, and 100 Hz, respectively, for the doublet acoustic (DA25, DA50, and DA100) (Sharma et al., 2022) and vibrotactile (DT25, DT50, and DT100) test trains (Ng et al., 2020). The cross-modal regular comparisons that spanned 12–44, 24–88, and 48–176 Hz for stimuli DA/DT25–100, respectively, were identical to experiment 1, regardless of sensory modality used for the comparison.

### Statistical analysis

The coefficient of determination (*R*^2^) of logit transformed psychophysics data was calculated to determine goodness of fit for the linear psychometric functions. A one-sample two-tailed *t*-test was performed to determine whether the experimentally obtained cross-modal PSEs differed significantly from the expected unimodal perceived frequency of the test stimulus. Within each experiment, a two-way repeated-measures ANOVA was used to examine whether the paradigm employed influenced the PSE. Similarly, a two-way repeated measures ANOVA analysed if presentation order of auditory and tactile stimuli in psychophysical trials affected PSEs. Tukey’s multiple comparisons tested mean Weber fractions across frequencies within a paradigm, and the paradigm effect on Weber fractions was analysed using a two-way repeated measures ANOVA. Unless specified, the data is presented as a mean with a 95% confidence interval.

## Results

### Perceptual equivalence between regular auditory and tactile stimuli (experiment 1)

Participants were able to make reliable comparisons of frequency across the two modalities as determined from the regression fits for the psychometric curves under both the auditory-tactile (*R*^2^ for logit-transformed psychophysical data, mean ± SD: 0.90 ± 0.07) and tactile-auditory (0.92 ± 0.06) paradigms.

**FIGURE 3:**
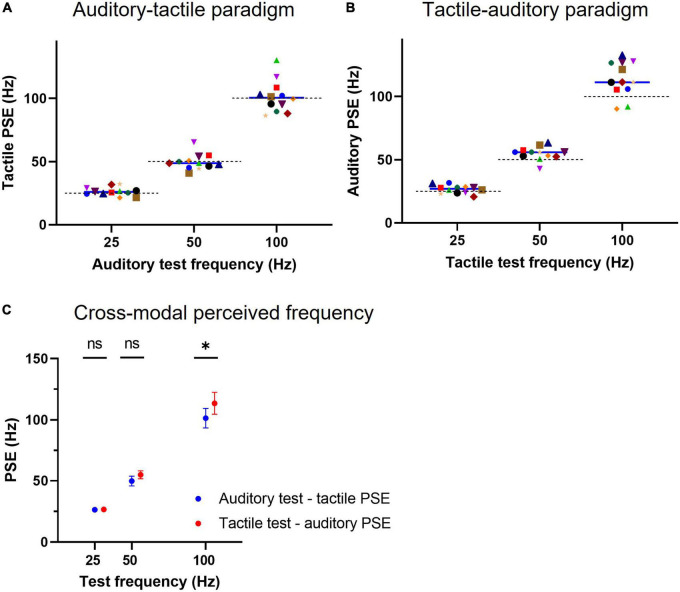
Cross-modal point of subjective equality (PSE) for regular tactile and auditory stimuli. **(A)** Individual subject PSEs obtained for auditory test conditions using tactile comparisons (auditory-tactile paradigm). A tactile PSE represents the frequency of a vibrotactile pulse train that is equally likely to be judged higher or lower than the auditory test stimulus. A solid line indicates a median PSE (*n* = 12) value for a test frequency, whereas a dashed line represents the expected unimodal PSE (or the physical frequency) of the test stimulus. **(B)** Individual subject PSEs for tactile test conditions using auditory comparisons (tactile-auditory paradigm), conventions as in panel **(A)**. **(C)** Cross-modal equivalent mean frequencies for both auditory and tactile stimuli of identical frequency, * represents *p* < 0.05 and error bars indicate ±95% CI.

The individual subject tactile PSEs for the auditory-tactile paradigm, where a test auditory stimulus was compared against a range of tactile frequencies, are illustrated in Figure 3A. The mean PSEs across subjects were not statistically different from the physical frequencies of the presented acoustic pulse trains: 26.3 (95% CI: 24.2–28.5) Hz for the 25 Hz test (*p* = 0.19, *n* = 12, one-sample two-tailed *t*-test); 49.8 (45.8–53.8) Hz for the 50 Hz test (*p* = 0.91, *n* = 12), and 101.4 (93.4–109.4) Hz for the 100 Hz (*p* = 0.70, *n* = 12) test stimulus. The physical frequency (or unimodal expected perceived frequency) of each test stimulus is represented by the dashed lines in the figure for comparison with the cross-modal obtained individual PSEs.

The individual subject auditory PSEs for the tactile-auditory paradigm are shown in Figure 3B. The auditory equivalent frequency (mean PSE) for the 25 Hz vibrotactile test was 26.6 (95% CI: 24.5–28.7) Hz, which was not different from its physical frequency (*p* = 0.12, *n* = 12, one-sample two-tailed *t*-test). The mean equivalent frequencies for the other two vibrotactile pulse trains: 54.9 (51.6–58.2) for the 50 Hz test and 113.6 (104.7–122.4) for the 100 Hz test did, however, differ from their corresponding test physical frequencies (*p* = 0.007 and *p* = 0.006, respectively, *n* = 12, one-sample two-tailed *t*-test).

Two-way repeated-measures ANOVA (test stimulus and cross-modal paradigm) on the data presented in Figure 3C indicated that choice of paradigm (auditory-tactile or tactile-auditory) accounts only for 0.7% of the total variation in PSEs [*F*(1, 11) = 5.147, *p* = 0.045], with test stimulus causing 93.3% [*F*(2, 22) = 1,062, *p* < 0.0001] and no interaction effect between factors. *Post-hoc* Šidák’s multiple comparisons test showed a statistical difference only between cross-modal mean PSEs of 100 Hz test stimulus (101.4 vs. 113.6 Hz, *p* = 0.01).

**FIGURE 4:**
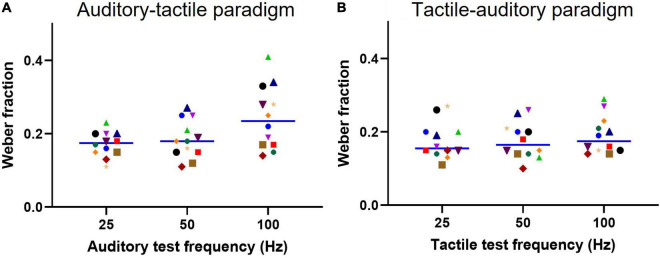
Weber fractions for each test stimulus. **(A)** Individual subject Weber fractions for each auditory test frequency using tactile comparisons (Auditory-tactile paradigm). **(B)** Similarly, individual subject Weber fractions for tactile test conditions using auditory comparisons (tactile-auditory paradigm). A solid line indicates a median (*n* = 12) Weber fraction value for a test frequency.

We also compared the discriminative ability of subjects when making cross-modal comparisons by calculating the Weber fraction. This value represents the sensitivity to changes in frequency. We plot the results in Figures 4A,B using the same conventions as for Figure 3. The mean Weber fractions for the auditory-tactile paradigm were 0.17 (95% CI: 0.15–0.19) for the 25 Hz test, 0.18 (0.15–0.21) for the 50 Hz test, and 0.24 (0.18–0.29) for the 100 Hz test stimulus. Tukey’s multiple comparisons test showed a significant difference between the 25 Hz and 100 Hz Weber fractions (*p* = 0.0146). The Weber fractions are similar across the three frequencies within the tactile-auditory paradigm: 0.17 (95% CI: 0.14–0.20) for the 25 Hz test; 0.17 (0.14–0.21) for the 50 Hz test, and 0.19 (0.15–0.22) for the 100 Hz test stimulus. A two-way repeated measures ANOVA (test frequency and paradigm) showed no significant effect of paradigm on Weber fractions [*F*(1.000, 11.00) = 4.484, *p* = 0.06] with test frequency accounting for 10.7% of the total variation [*F*(1.727, 19.00) = 6.300, *p* = 0.01]. The Weber fraction for a given test frequency was not statistically different across paradigms.

#### Presentation order of auditory and tactile stimuli does not influence point of subjective equality

To determine if the presentation order of a test stimulus in cross-modal frequency discrimination trials (consisting of audio-tactile pairs) alters the PSE, trials in which a test stimulus preceded the cross-modal comparisons and vice-versa were sorted for each subject in both the paradigms, then analysed separately for the corresponding PSEs. Each paradigm thus yielded two conditions, for example in the auditory-tactile paradigm: [A (test)–T (comparison)] and [T (comparison)–A (test)]. Each test stimulus was compared ten times against each of the six comparison stimuli to obtain PSEs.

**FIGURE 5:**
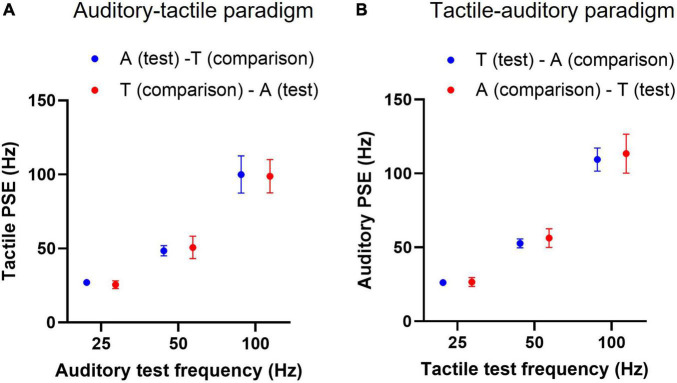
Cross-modal point of subjective equality (PSE) with test stimulus presentation order. **(A)** Mean tactile PSEs (*n* = 12) of an auditory test train when it precedes or follows the tactile comparisons in psychophysical frequency discrimination trials. “A (test)–T (comparison)” indicates an auditory test stimulus preceding the tactile comparison stimuli, and “T (comparison)–A (test)” denotes the opposite presentation order. **(B)** Similarly, mean auditory PSEs for a tactile test train at two conditions, conventions as in panel **(A)**. Error bars indicate ±95% CI.

The mean tactile PSEs (*n* = 12) of an auditory test stimulus, whether it precedes or follows the cross-modal tactile comparisons, were not statistically different; the presentation order accounted only for 0.0005% of total variation [*F*(1, 11) = 0.0018, *p* = 0.96, two-way RM ANOVA, Figure 5A]. Similarly, the order in which tactile tests were presented in the tactile-auditory paradigm had no effect on mean PSEs; tactile test order accounted for 0.13% of total variation in observed PSEs [*F*(1, 11) = 1.596, *p* = 0.23, two-way RM ANOVA, Figure 5B].

### Perceptual equivalence between cross-modal bursting and regular stimuli (experiment 2)

Having observed in Experiment 1 that cross-modal matching of equivalent frequencies was possible for regular test pulse trains, in the second set of experiments we explored if the same holds for more complex stimuli, such as trains of bursts consisting of a doublet of pulses. All participants could perform cross-modal frequency discrimination between bursting and regular stimuli in either auditory-tactile (mean psychometric function fit *R*^2^ ± SD: 0.89 ± 0.08) or tactile-auditory (*R*^2^ ± SD: 0.93 ± 0.05) paradigms, with similar ability to that observed for regular vs. regular cross-modal stimuli.

**FIGURE 6:**
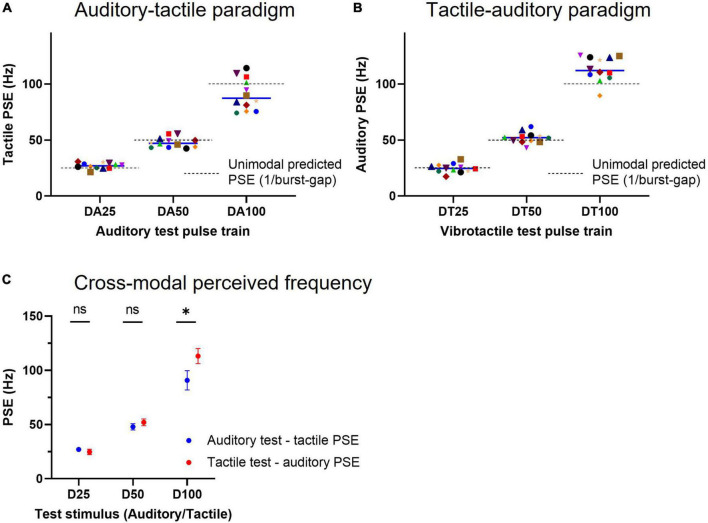
Cross-modal equivalent frequency for acoustic and tactile doublet-burst pulse trains. **(A)** Individual subject tactile PSEs obtained for acoustic test pulse trains schematically depicted in Figure 2A (Auditory-tactile paradigm). A tactile point of subjective equality (PSE) represents the frequency of a vibrotactile pulse train that is equally likely to be judged higher or lower than the acoustic test stimulus, indicating an equivalent frequency for a given acoustic train. Each solid line indicates median PSE (*n* = 12) values for test trains, while the dashed lines represent unimodal (i.e., test and comparisons from the same sensory modality) predicted PSEs estimated by the burst-gap model (1/burst-gap). **(B)** Individual subject auditory PSEs for vibrotactile test pulse trains shown in Figure 2A (Tactile-auditory paradigm), conventions as in panel **(A)**. **(C)** Cross-modal mean equivalent frequencies (*n* = 12) for both auditory and tactile pulse trains that are identical. * represents *p* < 0.05 and error bars denote ±95% CI.

The individual subject tactile PSEs for the three test acoustic doublet trains (schematically presented in Figure 2A), which vary both in burst rate/periodicity (23, 40, and 67 Hz) and mean pulse rate (46, 80, and 134 Hz, test trains DA25–DA100, respectively), are illustrated in Figure 6A. The mean equivalent tactile perceived frequency for stimulus DA25 was 27.0 Hz (95% CI: 25.3–28.8), for stimulus DA50 was 47.9 Hz (45.0–50.8) and stimulus DA100 was 90.9 Hz (81.9–99.8). Although these values are close to their burst-gap model predicted values depicted by the dashed lines [which correspond to the reciprocal of the inter-burst interval in the test train (Birznieks and Vickery, 2017; Ng et al., 2020, 2021)], the data for DA25 and DA100 differed from the predicted values of 25 Hz (*p* = 0.029, *n* = 12, one-sample *t*-test) and 100 Hz (*p* = 0.047, *n* = 12, one-sample *t*-test); while for stimulus DA50, there was no statistical difference from the burst-gap predicted value (50 Hz, *p* = 0.13, *n* = 12). However, the data for DA25 and DA100 was much more poorly predicted by their periodicity (23 Hz, *p* = 0.0004; and 67 Hz, *p* = 0.0001; one-sample *t*-test) or mean pulse rate (46 Hz, *p* < 0.0001; and 134 Hz, *p* < 0.0001) than from burst-gap predictions, suggesting the burst-gap model offers a better explanation.

When we determined auditory equivalent frequencies for the same patterns, we found 24.8 Hz (95% CI: 22.3–27.3) for stimulus DT25, 52.1 Hz (48.9–55.2) for stimulus DT50, and 113.2 Hz (106.2–120.3) for stimulus DT100 as shown in Figure 6B. The DT25 and DT50 PSEs matched the predicted values from the burst gap model of 25 Hz (*p* = 0.87, *n* = 12, one-sample *t*- test) and 50 Hz (*p* = 0.17). However, stimulus DT100 has a PSE higher than the stimulus burst-gap model predicted value of 100 Hz (*p* = 0.001, *n* = 12, one-sample *t*-test), yet this model predicted value remains a closer match to the observed PSE than mean pulse rate (134 Hz) and periodicity (67 Hz) predictions.

Two-way repeated measures ANOVA (test train and cross-modal paradigm) indicated that choice of paradigm accounts for 1.48% of the total variation [*F*(1, 11) = 17.88, *p* = 0.001], with maximum variation caused by the test train [90.74%, *F*(2, 22) = 469.4, *p* < 0.0001] and interaction between factors accounting only for 2.43% [*F*(2, 22) = 23.13, *p* < 0.0001]. *Post-hoc* Šidák’s multiple comparisons test showed a significant difference between cross-modal PSEs of stimulus D100 (90.87 vs. 113.2 Hz, *p* < 0.0001, Figure 6C).

## Discussion

We directly addressed the question of whether a perceptual equivalence of temporal frequency could be determined between auditory and tactile pulsatile stimuli for frequencies that span the flutter range up to the vibration range.

The first set of experiments explored the cross-modal perceptual equivalent frequency for regular auditory (auditory-tactile paradigm) and tactile (tactile-auditory paradigm) stimuli of 25, 50, and 100 Hz. The equivalent perceived tactile frequency for each auditory test train accurately matched its physical frequency, whereas the auditory equivalences for 50 and 100 Hz vibrotactile test pulse trains were marginally higher at 55 and 114 Hz, respectively. It may be that flutter and vibration are elaborated along different processing streams within each modality [as in touch (Talbot et al., 1968)], and show a close cross-modal match only in the flutter frequency stream (Romo and Salinas, 2003; Bendor and Wang, 2007) whereas the very different ranges between vibratory hum (up to 1 kHz) and pitch (up to 20 kHz) lead to a progressive mismatch in touch and hearing. This is also consistent with the results of Experiment 2 where tactile stimuli are matched with slightly higher auditory frequencies at 100 Hz, while there is close agreement at 25 and 50 Hz (Figures 6A,B).

We measured the Weber fractions for subjects performing our tasks, and found no difference between tactile-auditory and auditory-tactile paradigms. The mean value at 50 Hz of 0.18 across the two paradigms is a close match to our previously reported value for a straight tactile-tactile comparison at 40 Hz of 0.19 using the same pulsatile stimuli (Birznieks et al., 2019). This is an important observation, as it shows that not only are the perceived frequencies equivalent across the two sensory modalities, but that the ability to make cross-modal judgements is equally as good as that for a unimodal judgement.

The deviation of PSEs from the physical frequencies in the present study are within our calculated Weber fraction, and the Weber fraction of 0.2–0.3 reported in the literature for the vibrotactile stimulus (Goff, 1967; BensmaÏa et al., 2005; Li et al., 2018), which implies at most a small perceptual difference. As each auditory and tactile pulse was designed to elicit a single spike in respective activated afferents, the matched pulse trains would create similar temporal spike trains in the responding primary afferents. The close agreement in perceived frequencies across the two modalities suggest that inter-spike intervals in the spike trains are analysed analogously in higher centres of the two senses to extract similar frequency information.

We did not observe biases in the frequency discrimination tasks from the presentation sequence of tactile and auditory stimuli in the psychophysical trials that consisted of audio-tactile pairs. This finding is consistent with a previous study in which trained monkeys discriminated frequency between two sequentially presented auditory and tactile stimuli (Lemus et al., 2010). Notably, our findings also demonstrated that perceptual frequency interactions between touch and audition are not likely when the stimuli are separated in time. The finding that any given auditory or tactile stimulus elicited perceived frequency (measured in cross-modal psychophysical experiments using a range of frequencies) did not deviate from the expected unimodal perceived frequency, provides evidence that there was little interference present, with no cross-modal transfer of effects. However, for perceptual interactions that were reported between concurrently delivered auditory and tactile stimuli (Yau et al., 2009a,^2010^; Convento et al., 2019), the interactions may have arisen from a division of attention over both senses, pointing to the fact that audio-tactile interplay may require attentional binding that is cued by synchronicity (Schroeder et al., 2010; Convento et al., 2018).

We extended our study in the second set of psychophysical experiments to investigate the perceptual equivalence between cross-modal audio-tactile bursting (1 s periodic doublet train) and regular pulse trains, with the hypothesis that the inter-pulse interval of the cross-modal matched regular pulse train equates the burst gap interval in the bursting test stimulus, either auditory or tactile. Even though the stimulus pair of varying patterns (bursting vs. regular) was expected to evoke different perceptual qualities across two modalities (audition and touch) and was cross-matched to a different sense, subjects reliably discriminated the frequencies of cross-modal stimulus pairs, as indicated by the large *R*^2^ values (>0.89) of psychometric function fits applied to the psychophysical data. As hypothesised, the inter-pulse interval of the equated cross-modal regular pulse train was well-matched to the perceived frequency of the bursting stimuli in D25 and D50. The cross-modal PSEs for stimulus D100 (with 10 ms burst gap), though not precisely fitting to its burst gap prediction value, was better explained by the burst gap than the other properties of the test train such as the simple mean pulse rate or burst periodicity.

### Analogous temporal frequency processing mechanisms

The cerebral substrate underpinning the analysis of frequency signals is beyond the purview of this work, however, our findings make a strong case that the temporal frequency analysis mechanism is analogous across audition and touch, as the perceived frequency of doublet trains in either paradigm best corresponded to a unique temporal property—the duration of the burst-gap in test trains. Preliminary evidence from Nagarajan et al. (1998) suggests that temporal information processing may be mediated by common mechanisms in the auditory and tactile systems. Participants in their study were presented with pairs of vibratory tactile pulses and trained to distinguish the temporal interval between them. The findings revealed not just a drop in threshold as a function of training, but also that the better interval discrimination could be generalised to the auditory modality. Even though the generalisation was limited to an auditory base interval comparable to the one trained in touch, the results are noteworthy in that they show that temporal interval coding mechanisms could be shared centrally (Fujisaki and Nishida, 2010). Another study demonstrated that a prolonged exposure to acoustic stimuli (3 min adaptation) improves subsequent tactile frequency discrimination thresholds, and went as far as to propose that the two senses share or have overlapping neural circuits that facilitate basic frequency decoding (Crommett et al., 2017). In fact, the neural population that analogously signals flutter in cortices of both sensory modalities was identified [for somatosensory (Romo and Salinas, 2001, 2003) and auditory cortices (Lu et al., 2001; Bendor and Wang, 2007)], and an identical neuronal coding strategy was speculated to represent auditory and tactile flutter (Bendor and Wang, 2007; Saal et al., 2016). Furthermore, a recent study that used decoding analysis on brain activity discovered the common neural underpinnings of conscious perception between sensory modalities, including audition and touch (Sanchez et al., 2020). It has been suggested that the inner ear evolved as a highly frequency-specific responder from the skin, and the tactile system expanded the spectrum of low-frequency hearing (Fritzsch et al., 2007).

Others have argued for analogous processing mechanisms across senses for other properties; higher-order representations—of object shape and motion—were also found to be strikingly analogous in touch and vision (Pei et al., 2008; Yau et al., 2009b,^2016^). The coding similarities across modalities provide evidence for the existence of canonical computations: the nervous system seems to have evolved to implement similar computations across modalities to extract similar information about the environment, regardless of the source of inputs (Pack and Bensmaia, 2015; Crommett et al., 2017).

### Cross-modal perceptual judgements

When subjects discriminate the difference in frequency between two sequentially applied stimuli, the discrimination task can be thought of as a chain of neural operations that include encoding information of the two successive stimuli, storing the first stimulus in working memory, comparing the second stimulus to the memory trace left by the first stimulus, and finally communicating the result (Romo and Salinas, 2001; Hernández et al., 2002).

The fact that perceptual equivalence of frequency could be achieved not only between cross-modal stimuli with closely matching afferent temporal firing patterns (regular vs. regular), but also between those with varying patterns (regular vs. bursting) that have different underlying spike patterns, suggests that the audio-tactile cross-modal perceptual decision might occur central to the primary sensory cortices—as they decode only information for their individual modality and do so only during the stimulus presentation period, and not during the delay between the two stimuli (Romo et al., 2002; Lemus et al., 2010). The neurons of the primary somatosensory cortex (S1) do not engage in the working memory component of the task, nor do they compare the difference between the two input frequencies (Hernández et al., 2000). Therefore, the cross-modal perceptual decision or convergence of two distinct processing channels must occur outside these cortical areas (Fuster et al., 2000; Schroeder and Foxe, 2002).

The intriguing question is where and how the modality-specific information is transformed into a supramodal signal that allows for cross comparison. Are these two sensory modalities encoded in working memory by the same group of neurons? For example, the neurons in the pre-supplementary motor area, an area shown to participate in perceptual judgements (Tanji, 1994; Hernández et al., 2002; Hernandez et al., 2010), were found to encode tactile and acoustic frequency information in working memory using the exact representation for both modalities (Vergara et al., 2016). Similarly, the medial premotor cortex was also shown to contribute to supramodal perceptual decision making (Haegens et al., 2017). Thus, single neurons from the frontal cortical region are good candidates to encode more than one sensory modality contributing to multimodal processing during perceptual judgements. Such supramodal representations may be quite restricted, as it may exist only for task parameters such as stimulus frequency that are strongly congruent and similarly discriminable across modalities (tactile and acoustic) (Vergara et al., 2016). Nevertheless, the comparison of stored and ongoing sensory information has been reported to occur widely—no single location can be designated as the unique locus of decision making (Romo and Salinas, 2003).

### Methodological implications for future psychophysics research

Psychophysics is an essential tool in neuroscience to investigate underlying neural mechanisms for sensory perceptions. It must have a comparison standard that can be reliably and consistently equated to the test stimulus of interest (Laming and Laming, 1992). Conventionally, comparisons have been established using standardised stimuli in the same modality as the test stimuli of interest. But as the complexity of enquiries into human frequency perception advances, the same-modality comparison standard becomes challenging as an increasing number of confounders for frequency, such as varying stimulus intensities, stimulation sites and several stimulating electrodes (McIntyre et al., 2016) are introduced into research designs. Our findings of cross-modal matching of stimuli at identical frequencies raise the possibility of employing a cross-modal comparison standard for future psychophysical investigations in the two modalities, thereby overcoming the prevailing methodological limitations. Jelinek and McIntyre’s (2010) work provides an insight, as they used auditory noises of varied volumes to assess the perceived intensities of tactile stimuli. Similarly, the method of cross-modality matching to compare the perceived magnitude of electrical stimulation on the abdomen to that of a 500 Hz tone has been deployed (Sachs et al., 1980). In early pioneering work, von Békésy (1957, 1959) had already flagged that the sensation of touch may be used as a model for studying functional features of hearing (see also Gescheider, 1970). As stimulation properties (except frequency) in the auditory system have different coding schemes to their counterparts in the tactile system (Chatterjee and Zwislocki, 1997; Bollini et al., 2021), tactile confounders for vibrotactile frequency are less likely to bias pitch perception of auditory stimuli and vice versa. The perceived frequencies of tactile stimuli applied at different locations on the skin or with different shaped probes could all be matched to auditory stimuli of equivalent perceived pitch, allowing standardisation across different labs, and enabling these results to be compared in absolute terms regardless of skin location or probe shape.

## Conclusion

Identical acoustic and vibrotactile pulse trains of simple and complex temporal features produce equivalent perceived frequencies with precise accuracy within the flutter range. This applied even for temporally-complex stimuli, indicating analogous frequency computation mechanisms deployed in higher centres, which supports the notion of a canonical computation. The findings suggest new experimental possibilities where the perceived frequency elicited by tactile stimuli, either regular or complex, within the flutter range can be measured explicitly using cross-modal auditory comparisons, and vice versa.

## Data availability statement

The original contributions presented in this study are included in the article/supplementary material, further inquiries can be directed to the corresponding author.

## Ethics statement

The studies involving human participants were reviewed and approved by Human Research Ethics Committee UNSW Sydney. The patients/participants provided their written informed consent to participate in this study.

## Author contributions

DS performed the experiments, analysed the data, drafted the manuscript, and prepared the figures. DS, IB, and RV interpreted the experimental results. DS, KN, IB, and RV conceived and designed the study, edited and revised the manuscript, and approved the final submitted version.
